# *Immp2l* Deficiency Induced Granulosa Cell Senescence Through STAT1/ATF4 Mediated UPR^mt^ and STAT1/(ATF4)/HIF1α/BNIP3 Mediated Mitophagy: Prevented by Enocyanin

**DOI:** 10.3390/ijms252011122

**Published:** 2024-10-16

**Authors:** Xiaoya Qu, Pengge Pan, Sinan Cao, Yan Ma, Jinyi Yang, Hui Gao, Xiuying Pei, Yanzhou Yang

**Affiliations:** School of Basic Medical Science, Key Laboratory of Fertility Preservation and Maintenance of Ministry of Education, Ningxia Medical University, Yinchuan 750004, China; quxiaoya0504@163.com (X.Q.); 20160120@nxmu.edu.cn (P.P.); csn2438690257@163.com (S.C.); nxmumayan@163.com (Y.M.); jinyi1852021@163.com (J.Y.); gaohui@nxmu.edu.cn (H.G.)

**Keywords:** cell senescence, STAT1, UPR^mt^, mitophagy, enocyanin, HIF1α/BNIP3 pathway

## Abstract

Dysfunctional mitochondria producing excessive ROS are the main factors that cause ovarian aging. *Immp2l* deficiency causes mitochondrial dysfunction and excessive ROS production, leading to ovarian aging, which is attributed to granulosa cell senescence. The pathway controlling mitochondrial proteostasis and mitochondrial homeostasis of the UPR^mt^ and mitophagy are closely related with the ROS and cell senescence. Our results suggest that *Immp2l* knockout led to granulosa cell senescence, and enocyanin treatment alleviated *Immp2l* deficiency-induced granulosa cell senescence, which was accompanied by improvements in mitochondrial function and reduced ROS levels. Interestingly, redox-related protein modifications, including S-glutathionylation and S-nitrosylation, were markedly increased in *Immp2l*-knockout granulosa cells, and were markedly reduced by enocyanin treatment. Furthermore, STAT1 was significantly increased in *Immp2l*-knockout granulosa cells and reduced by enocyanin treatment. The co-IP results suggest that the expression of STAT1 was controlled by S-glutathionylation and S-nitrosylation, but not phosphorylation. The UPR^mt^ was impaired in *Immp2l*-deficient granulosa cells, and unfolded and misfolded proteins aggregated in mitochondria. Then, the HIF1α/BNIP3-mediated mitophagy pathway was activated, but mitophagy was impaired due to the reduced fusion of mitophagosomes and lysosomes. The excessive aggregation of mitochondria increased ROS production, leading to senescence. Hence, Enocyanin treatment alleviated granulosa cell senescence through STAT1/ATF4-mediated UPR^mt^ and STAT1/(ATF4)/HIF1α/BNIP3-mediated mitophagy.

## 1. Introduction

Female fertility is reduced or impaired by accelerated ovarian aging, and mitochondrial dysfunction is one of the hallmarks of aging. Mitochondrial dysfunction drives ovarian aging [1].

Mitochondrial dysfunction can induce cell senescence due to excessive ROS (Reactive Oxygen Species) production [2], and mitochondrial proteostasis and mitochondrial homeostasis are controlled by the mitochondrial quality control (MQC) system, which includes the mitochondrial unfolded protein response (UPR^mt^) and mitophagy. Mitophagy helps remove partially damaged mitochondria to ensure that mitochondria are healthy and functional, and the overactivation or inhibition of the UPR^mt^ can even accelerate abnormal energy metabolism and mitochondrial dysfunction-induced cell senescence. Thus, an imbalance in MQC can accelerate cellular senescence and aging, and appropriate interventions in MQC can delay the aging process and extend the lifespan [3].

Signal transducer and activator of transcription 1 (*STAT1*) is a nuclear transcription factor that regulates genes involved in the cell cycle, cell survival and the immune response. Despite being activated downstream of common cytokines and growth factors, *STAT1* plays antagonistic roles, and a disruption of this balance or phosphorylation levels shifts cells from survival to apoptotic cell death or from inflammatory to anti-inflammatory responses [4]. STAT proteins are latent in the cytoplasm of resting cells as monomers or unphosphorylated dimers until they are activated by growth factors and cytokines that bind to a specific cell receptor [4]. Aside from phosphorylation, STAT activity can be regulated by other posttranslational modifications, such as ubiquitination, acetylation, methylation, S-glutathionylation and ISGylation [4]; in particular, redox regulates STAT1, and redox-related protein posttranslational modifications, including S-glutathionylation [5] and S-nitrosylation [6], are closely related to posttranslational modifications of STAT1 [7]. In addition, STATs proteins, including STAT1, have been proven to localize in the mitochondria, and a deficiency of STAT1 protein in mitochondria is closely related with mitochondrial dysfunction, such as an imbalance of mitochondrial biogenesis and mitochondrial homeostasis. However, the STAT1 protein involved in maintaining the mitochondrial function is largely unknown, and whether UPR^mt^ and mitophagy are regulated by STAT1 is unclear.

Our previous study suggested that ovarian aging in mice was caused by a deficiency in inner mitochondrial membrane peptidase 2-like (Immp2l), which is associated with the accelerated production of excessive ROS, then leading to granulosa cell senescence [8]. Whether the STAT1 protein was regulated by ROS-related posttranslational modifications, including S-glutathionylation and S-nitrosylation, and whether UPR^mt^ and mitophagy regulated by STAT1 in *Immp2l* deficiency induced granulose cell senescence, is unknown.

Anthocyanidins, which are a class of water-soluble flavonoids, have been reported to possess several biological activities, including antioxidative, anti-inflammatory, and anticancer activities [9], and anthocyanidins can be extracted from different plant sources, such as fruits, flowers and vegetables; dietary sources of anthocyanidins include red and purple berries, grapes, apples, plums and cabbage [10]. Several studies have suggested that anthocyanidins play important roles in human health and that anthocyanidins are known for their therapeutic effects on many human diseases, such as cardiovascular and neurodegenerative diseases [10], obesity-induced inflammation [11], tension glaucoma [12], cancer [13], aging and cell senescence [14,15]. Enocyanin is a novel identified anthocyanidins, and it is extracted from grapes, but the role of enocyanin is largely unknown, especially in STAT1-regulated UPR^mt^, and that of mitophagy in *Immp2l* deficiency-induced granulose cell senescence is unclear.

Therefore, in this work, the role of STAT1-mediated UPR^mt^ and mitophagy, as well as the role of enocyanin in *Immp2l* deficiency-induced granulosa cell senescence, were investigated. This study provides novel insights into and evidence of the role of STAT1 in maintaining mitochondrial function through UPR^mt^ and mitophagy, as well as that of enocyanin in cell senescence, and reveals a novel target for alleviating mitochondrial dysfunction-induced granulosa cell senescence and ovarian aging.

## 2. Results

### 2.1. Impaired UPR^mt^ and Mitophagy Caused by Immp2l Knockdown Induced Primary Granulosa Cell Senescence and Mitigated by Enocyanin

**Figure 1:**
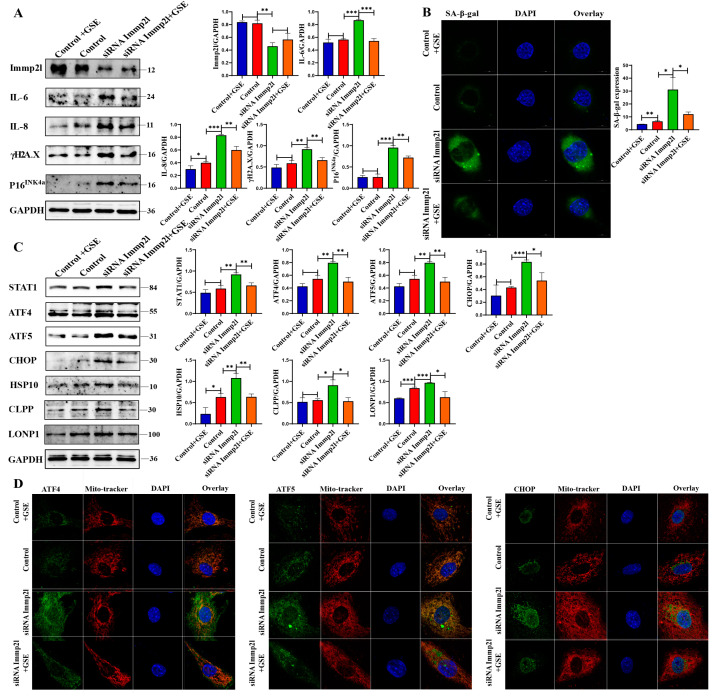

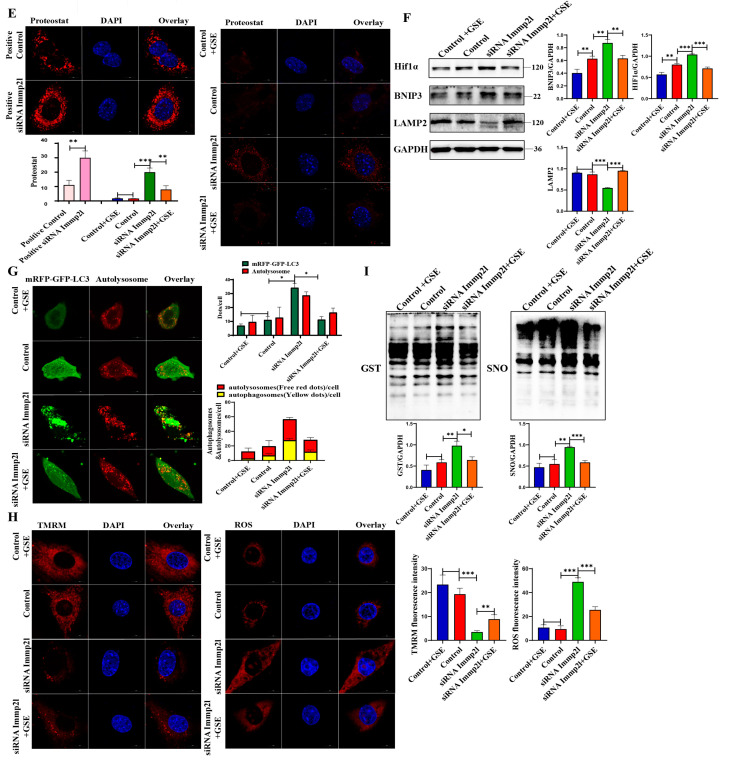
Enocyanin delays *Immp2l* knockdown-induced primary granulosa cell senescence. (**A**) Western blot analysis of cell senescence marker molecules in *Immp2l*-knockdown granulosa cells treated with enocyanin. (**B**) Staining of senescence-associated β-galactosidase in *Immp2l*-knockdown granulosa cells treated with enocyanin, at least 50 different cells in different fields were analyzed, and the dots were blindly counted by three different individuals. (**C**) Western blot analysis of cell STAT1 and UPR^mt^ molecules in *Immp2l* knockdown granulosa cells treated with enocyanin. (**D**) Detection of the colocalization of UPR^mt^ molecules ATF4, ATF5, CHOP and Mito-tracker. (**E**) Detection of protein aggresomes with the PROTEOSTAT Aggresome Detection Kit in *Immp2l*-knockdown granulosa cells treated with enocyanin. (**F**) Western blot analysis of mitophagy molecules and lysosome membrane protein Lamp2 in *Immp2l*-knockdown granulosa cells treated with enocyanin. (**G**) Detection of autophagic flux with the mCherry-GFP-LC3 lentivirus in *Immp2l*-knockdown granulosa cells treated with enocyanin; at least 50 different cells in different fields were analyzed, and the dots were blindly counted by three different individuals. (**H**) Detection of mitROS and mitochondrial membrane potential in *Immp2l*-knockdown granulosa cells treated with enocyanin. (**I**) Western blot analysis of S-glutathionylation and S-nitrosylation in *Immp2l*-knockdown granulosa cells treated with enocyanin. (* *p* < 0.05; ** *p* < 0.01; *** *p* < 0.001), (Scale bar: 10 μm).

*Immp2l*-knockout mice were born, and limited granulosa cells were isolated from them in order to confirm the role of enocyanin in anti-aging. Primary granulosa cells were treated with enocyanin in vitro, and the results suggest that senescence was alleviated in enocyanin-treated *Immp2l* knockdown granulosa cells, which was accompanied by a significant reduction in the senescence markers IL-6, IL-8, γH2AX and P16^INK4a^ (Figure 1A), which results were further supported by the staining of senescence-associated β-galactosidase (Figure 1B). In addition, the UPR^mt^ and mitophagy were restored in enocyanin-treated *Immp2l* knockdown granulosa cells, which was accompanied by significant decreases in the STAT1 and UPR^mt^ markers ATF4, ATF5, CHOP, HSP60, HSP10, CLPP and LONP1 (Figure 1C) and the mitophagy markers HIF1α and BNIP3, as well as significant increases in lysosome marker Lamp2 (Figure 1F). These results are further supported by results from the colocalization of ATF4, ATF5, CHOP and Mito-tracker (Figure 1D), as well as results from the detection of protein aggresomes (Figure 1E), autophagic flux (Figure 1G) and mitochondrial function (mitROS levels and MMP levels) (Figure 1H). Additionally, S-glutathionylation and S-nitrosylation were significantly decreased in enocyanin-treated *Immp2l*-knockdown granulosa cells (Figure 1I). Therefore, these results suggest that the impairment of UPR^mt^ and mitophagy is closely related with the ROS-induced primary granulosa cell senescence in *Immp2l*-knockout primary granulosa cells, and improve UPR^mt^ and mitophagy by enocyanin alleviated ROS level.

In addition, the ovaries from 28 days old mice were collected according to our previous study [8], and our results reveal that the three UPR^mt^ core molecules ATF4, ATF5 and CHOP were all aggregated in the cytoplasm but not translocated into the nucleus in granulosa cells in the secondary follicle in *Immp2l*-knockout mice (Figure S1), and the UPR^mt^ was impaired in senescent granulosa cells in secondary follicles in vivo.

### 2.2. Enocyanin Alleviates Granulosa Cell Senescence and Improves Oxidative Damage Induced by UPR^mt^ and Mitophagy

**Figure 2:**
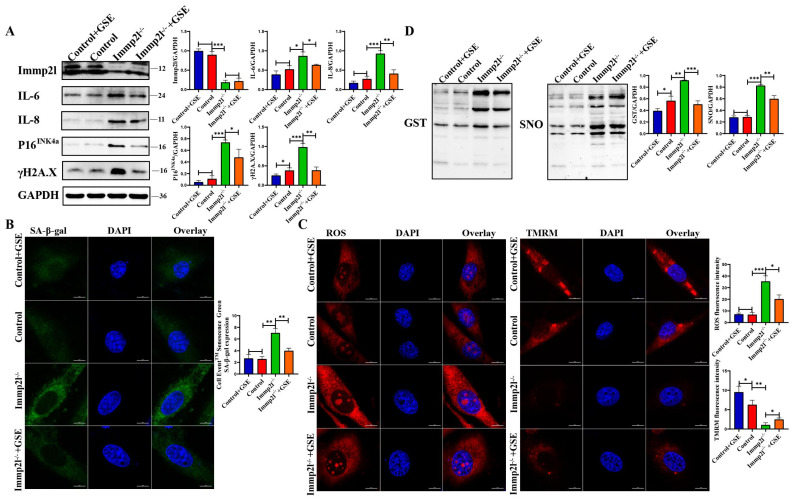
Enocyanin delays *Immp2l* deficiency-induced granulosa cell senescence. (**A**) Western blot analysis of cell senescence marker molecules in *Immp2l*-deficient granulosa cells treated with enocyanin. (**B**) Staining of senescence-associated β-galactosidase in *Immp2l*-deficient granulosa cells treated with enocyanin; at least 50 different cells in different fields were analyzed, and the dots were blindly counted by three different individuals. (**C**) Detection of mitROS and mitochondrial membrane potential in *Immp2l*-deficient granulosa cells treated with enocyanin. (**D**) Western blot analysis of S-glutathionylation and S-nitrosylation in Immp2l-deficient granulosa cells treated with enocyanin. (* *p* < 0.05; ** *p* < 0.01; *** *p* < 0.001), (Scale bar: 10 μm).

The KGN cells was employed to knock out *Immp2l* gene, and the Western blot results suggest that the knockout of the *Immp2l* gene (*Immp2l*^−/−^) in the human granulosa cell line KGN was successful, and the protein expression of the senescence markers IL-6, IL-8, γH2AX and P16^INK4a^ was significantly increased in *Immp2l*^−/−^ granulosa cells compared with the control (*Immp2l*^+/+^); therefore, granulosa cell senescence was triggered by *Immp2l* gene deficiency, and senescence was mitigated by enocyanin treatment, which was accompanied by a significant decrease in the senescence markers IL-6, IL-8, γH2AX and P16^INK4a^ in enocyanin-treated *Immp2l*^−/−^ granulosa cells compared with *Immp2l*^−/−^ granulosa cells (Figure 2A). These results were further supported by the staining of senescence-associated β-galactosidase (Figure 2B). Immp2l deficiency causes granulosa cell senescence, and granulosa cell senescence is mitigated by enocyanin treatment. In addition, KGN cells shared the same traits with primary granulosa cells in ROS-induced granulosa cells.

Cell senescence is closely related to mitochondrial dysfunction and ROS production [16]; therefore, mitochondrial function, including mitROS and mitochondrial membrane potential (MMP), was examined, and the results suggest that mitochondrial function was impaired, accompanied by significantly increased mitROS levels and decreased MMP in *Immp2l*^−/−^ granulosa cells compared with the control. Enocyanin restored mitochondrial function, markedly decreased mitROS levels and increased MMP in enocyanin-treated *Immp2l*^−/−^ granulosa cells compared with *Immp2l*^−/−^ granulosa cells (Figure 2C). Then, S-glutathionylation and S-nitrosylation were detected in redox-related proteins, and the results suggest that S-glutathionylation (GST) and S-nitrosylation (SNO) levels were significantly increased and significantly decreased, respectively, by enocyanin treatment in *Immp2l*^−/−^ granulosa cells (Figure 2D). Thus, mitochondrial dysfunction in granulosa cells induced by *Immp2l* deficiency was improved by enocyanin treatment.

### 2.3. STAT1 Was Regulated by GST and SNO but Not Phosphorylation

**Figure 3:**
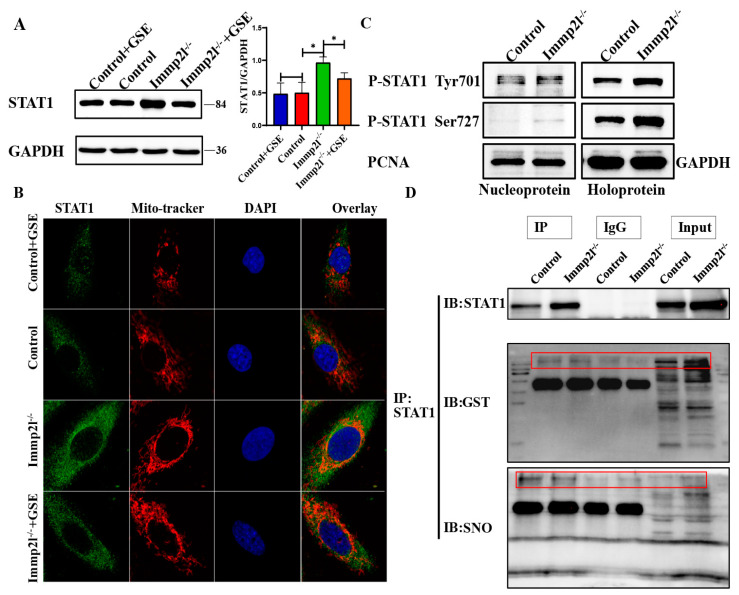
The translational modification of GST and SNO but not phosphorylation regulates STAT1. (**A**) Western blot analysis of STAT1 in *Immp2l*-deficient granulosa cells treated with enocyanin. (**B**) Colocalization of STAT1 and Mito-tracker in *Immp2l*-deficient granulosa cells treated with enocyanin. (**C**) Western blot analysis of phosphorylated STAT1 in *Immp2l*-deficient granulosa cells. (**D**) Co-IP confirmation of the interaction of STAT1 and S-glutathionylation and S-nitrosylation; at least 50 different cells in different fields were analyzed, and the dots were blindly counted by three different individuals. (* *p* < 0.05), (Scale bar: 10 μm).

Previous studies suggested that ROS induces STAT1 [17], and our Western blot results suggest that STAT1 was markedly increased in *Immp2l*^−/−^ granulosa cells compared with the control, while STAT1 was markedly reduced in enocyanin-treated *Immp2l*^−/−^ granulosa cells compared with *Immp2l*^−/−^ granulosa cells (Figure 3A). The STAT1 protein can be phosphorylated and translocate into the nucleus [18], and STAT1 is also regulated through transcriptional modification by S-glutathionylation and S-nitrosylation [5,6,7]. STAT1 was localized in the mitochondria and aggregated in the mitochondria of *Immp2l*-deficient granulosa cells, while the STAT1 protein’s accumulation in mitochondria was reduced by enocyanin treatment (Figure 3B). Consistently, phosphorylated STAT1 (Tyr701, Ser727) protein levels were not increased in the nucleus in *Immp2l*^−/−^ granulosa cells (Figure 3C). Thus, the STAT1 protein was not posttranslationally modified by phosphorylation, and further Co-IP results show that the STAT1 protein was posttranslationally modified by S-glutathionylation and S-nitrosylation (Figure 3D).

### 2.4. Enocyanin Decrease Immp2l Deficiency-Induced UPR^mt^ and Mitophagy

**Figure 4:**
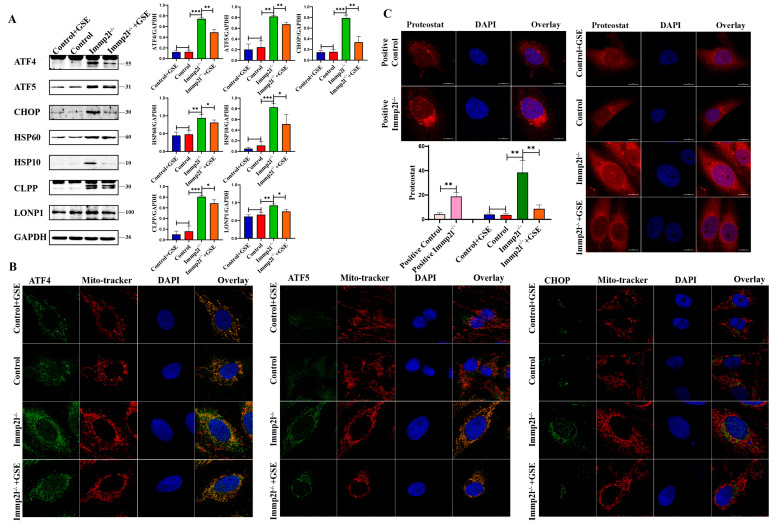
UPR^mt^ pathway proteins were altered in *Immp2l*-deficient granulosa cells and regulated by enocyanin. (**A**) Western blot analysis of UPR^mt^ marker molecules in *Immp2l*-deficient granulosa cells treated with enocyanin. (**B**) Colocalization of the UPR^mt^ molecules ATF4, ATF5, CHOP and Mito-tracker in *Immp2l*-deficient granulosa cells treated with enocyanin. (**C**) Detection of protein aggresomes with the PROTEOSTAT Aggresome Detection Kit in *Immp2l*-deficient granulosa cells treated with enocyanin. (* *p* < 0.05; ** *p* < 0.01; *** *p* < 0.001) (scale bar: 10 μm).

Mitochondrial proteostasis and mitochondrial homeostasis are maintained by the UPR^mt^ [19,20] and mitophagy [21]. Mitochondrial dysfunction might be attributed to dysfunctional UPR^mt^ and mitophagy. Our results reveal that the UPR^mt^ marker proteins ATF4, ATF5, CHOP, HSP60, HSP10, CLPP and LONP1 were significantly increased (Figure 4A), but ATF4, ATF5 and CHOP did not translocate into the nucleus and accumulate in mitochondria (Figure 4B). Thus, UPR^mt^ dysfunction occurred in *Immp2l*-knockout granulosa cells, and UPR^mt^ impairment was alleviated by enocyanin treatment in *Immp2l*^−/−^ granulosa cells accompanied with decreased UPR^mt^ marker proteins and reduced accumulations of ATF4, ATF5 and CHOP proteins in mitochondria (Figure 4A,B). Indeed, mitochondrial proteostasis was impaired by *Immp2l* deficiency, and mitochondrial proteostasis was restored by enocyanin treatment. This result was further supported by the detection of protein aggresomes (Figure 4C).

**Figure 5:**
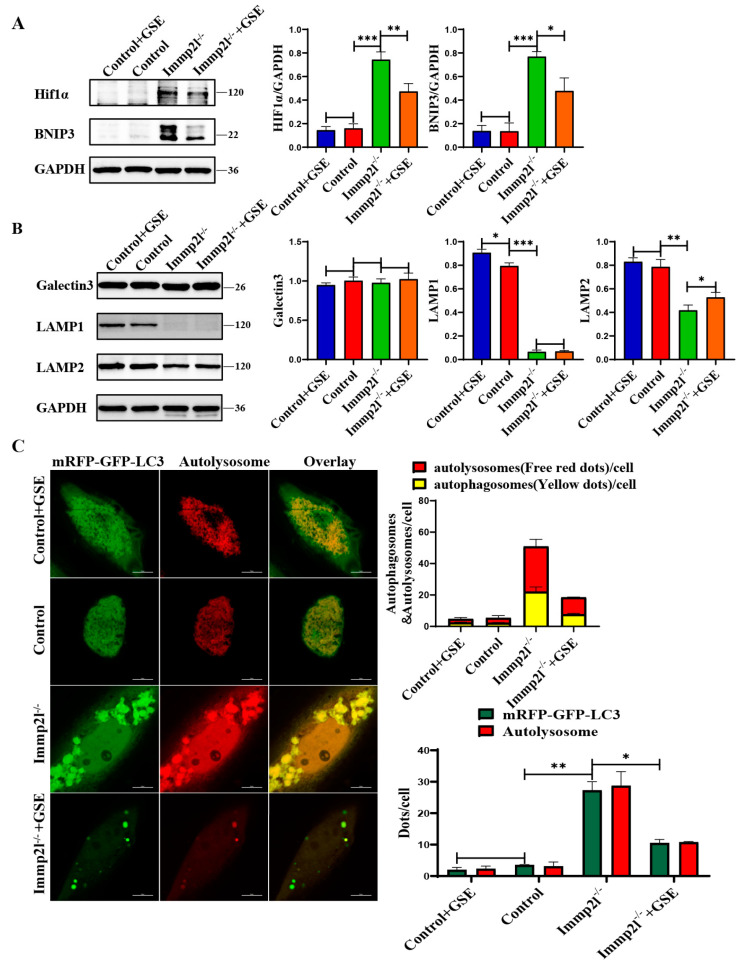
Mitophagy pathway proteins are altered in *Immp2l*-deficient granulosa cells and regulated by enocyanin. (**A**) Western blot analysis of mitophagy marker molecules in *Immp2l*-deficient granulosa cells treated with enocyanin. (**B**) Western blot analysis of the lysosome marker molecules Lamp1, Lamp2 and Galectin3 in *Immp2l*-deficient granulosa cells treated with enocyanin. (**C**) Detection of autophagic flux with the mCherry-GFP-LC3 lentivirus in *Immp2l*-deficient granulosa cells treated with enocyanin; at least 50 different cells in different fields were analyzed, and the dots were blindly counted by three different individuals. (* *p* < 0.05; ** *p* < 0.01; *** *p* < 0.001) (scale bar: 10 μm).

UPR^mt^ impairment leads to unfolded and misfolded protein accumulation in mitochondria, resulting in mitochondrial dysfunction and aggregation in cells, and mitophagy is activated to clear aggregated mitochondria and proteins [22]. The HIF1α-BNIP3 mitophagy pathway plays an important role in maintaining mitochondrial homeostasis [23], and the HIF1α-BNIP3–mitophagy pathway is impaired in the ovarian granulosa cells of PCOS rats [24]. Thus, the role of the HIF1α-BNIP3-mitophagy pathway in *Immp2l* deficiency-induced senescence in granulosa cells was explored, and the results reveal that HIF1α and BNIP3 were significantly increased in *Immp2l*^−/−^ granulosa cells and decreased by enocyanin treatment (Figure 5A). The lysosomal-associated membrane protein 2 (Lamp2) was significantly decreased in *Immp2l*^−/−^ granulosa cells and increased by enocyanin treatment, while lysosomal-associated membrane protein 1 (Lamp1) and lysosome damage marker protein Galectin3 [25] were not altered by enocyanin treatment (Figure 5B). Therefore, the activated HIF1α/BNIP3-mediated mitophagy and markedly decreased Lamp2 levels indicate an imbalance in the mitophagy–lysosome degradation axis due to the impaired fusion of mitophagosomes and lysosomes, and restored the balance in the mitophagy–lysosome degradation axis attribute to increased Lamp2 levels in enocyanin-treated *Immp2l*^−/−^ granulosa cells (Figure 5B). In addition, aggregated autophagosomes were markedly increased in *Immp2l*^−/−^ granulosa cells and decreased by enocyanin treatment, as shown by mCherry-GFP-LC3 staining (Figure 5C). Thus, the UPR^mt^ and HIF1α/BNIP3 mitophagy pathways were impaired in *Immp2l*^−/−^ granulosa cells and subsequently restored by enocyanin.

### 2.5. UPR^mt^ and Mitophagy Are Regulated by STAT1 Inhibition, Which Is Accompanied by the Alleviation of Granulosa Cell Senescence

**Figure 6:**
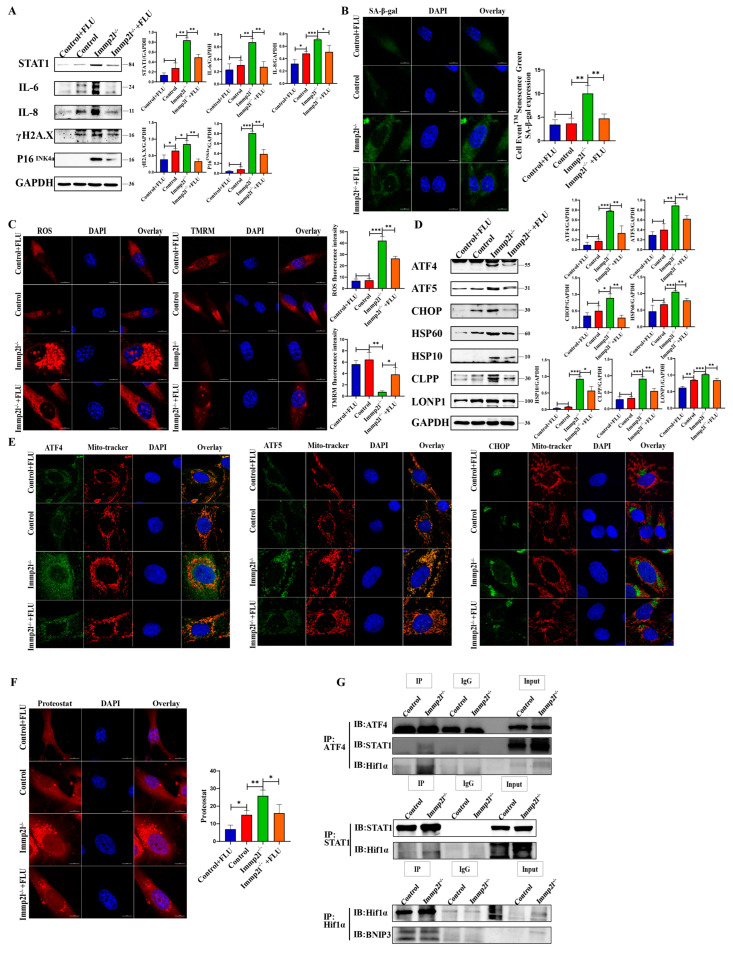
The UPR^mt^ and senescence proteins regulated by the STAT1 inhibitor fludarabine (FLU). (**A**) Western blot analysis of cell senescence marker molecules in *Immp2l*-deficient granulosa cells treated with the STAT1 inhibitor fludarabine (FLU). (**B**) Staining of senescence-associated β-galactosidase in *Immp2l*-deficient granulosa cells treated with the STAT1 inhibitor fludarabine (FLU); at least 50 different cells in different fields were analyzed, and the dots were blindly counted by three different individuals. (**C**) Detection of mitROS and mitochondrial membrane potential in *Immp2l*-deficient granulosa cells treated with the STAT1 inhibitor fludarabine (FLU); at least 50 different cells in different fields were analyzed, and the dots were blindly counted by three different individuals. (**D**) Western blot analysis of UPR^mt^ marker molecules in *Immp2l*-deficient granulosa cells treated with the STAT1 inhibitor fludarabine (FLU). (**E**) Colocalization of the UPR^mt^ marker molecules ATF4, ATF5, CHOP and Mito-tracker. (**F**) Detection of protein aggresome with the PROTEOSTAT Aggresome Detection Kit; at least 50 different cells in different fields were analyzed, and the dots were blindly counted by three different individuals. (**G**) Co-IP detection and analysis of the interaction of UPR^mt^ ATF4 with STAT1, ATF4 and HIF1α, HIF1α and BNIP3, STAT1 and HIF1α. (* *p* < 0.05; ** *p* < 0.01; *** *p* < 0.001), (scale bar: 10 μm).

To explore the effect of STAT1 on granulosa cell senescence, the STAT1 inhibitors fludarabine (FLU) and siRNA were used to inhibit and knock down *STAT1*, respectively, and the results show that STAT1, IL-6, IL-8, γH2AX and P16^INK4a^ were significantly decreased in STAT1-inhibited *Immp2l*^−/−^ granulosa cells compared with *Immp2l*^−/−^ granulosa cells (Figure 6A and Figure S2). These results are further supported by the results of the staining of senescence-associated β-galactosidase (Figure 6B). Inhibiting STAT1 restored mitochondrial function, markedly decreased mitROS levels and increased MMP in *Immp2l*^−/−^ granulosa cells (Figure 6C). Therefore, inhibiting STAT1 alleviates granulosa cell senescence.

In addition, the UPR^mt^ markers ATF4, ATF5, CHOP, HSP60, HSP10, CLPP and LONP1 were significantly decreased in STAT1-inhibited *Immp2l*^−/−^ granulosa cells compared with *Immp2l*^−/−^ granulosa cells (Figure 6D and Figure S3). The colocalization of ATF4, ATF5, CHOP and mitochondria (Figure 6E) with protein aggresomes (Figure 6F) was markedly decreased in STAT1-inhibited *Immp2l*^−/−^ granulosa cells compared with *Immp2l*^−/−^ granulosa cells. Furthermore, the co-IP results reveal that ATF4, which is a core molecule in the UPR^mt^, is regulated by STAT1 (Figure 6G). Therefore, the core molecule ATF4 in UPR^mt^ is regulated by the STAT1.

**Figure 7:**
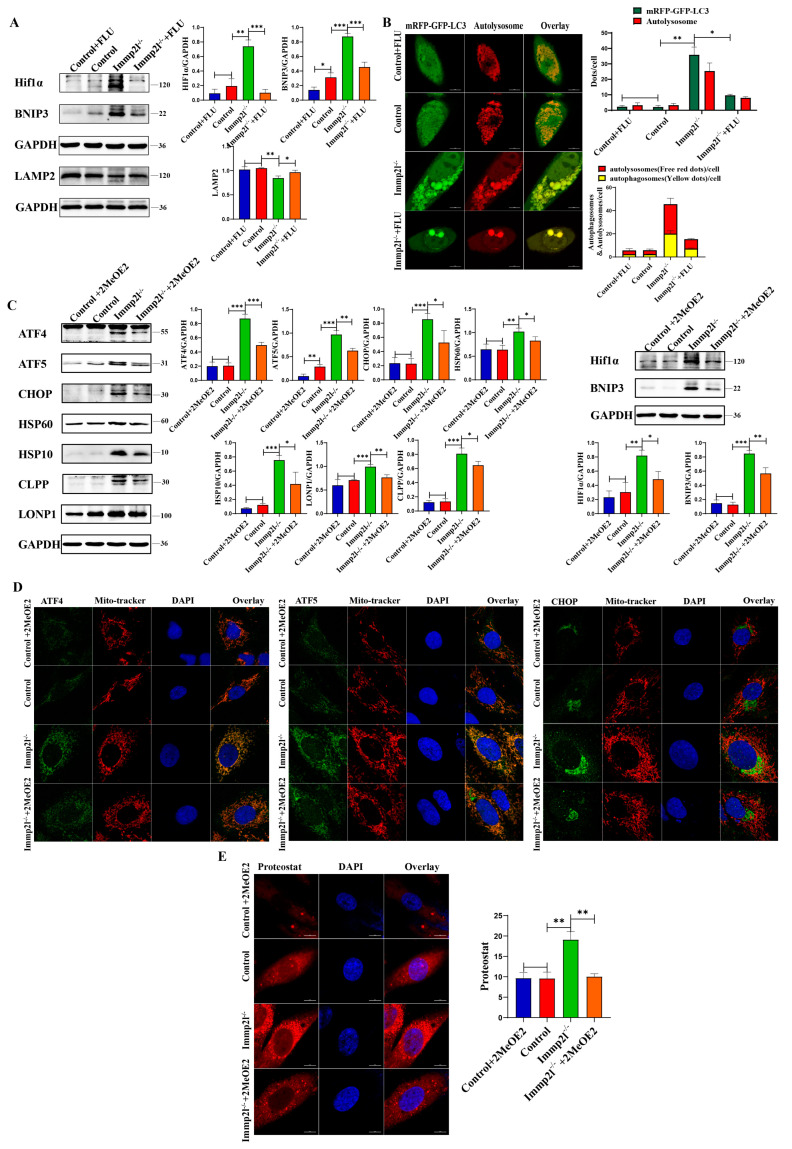
Mitophagy and lysosomal proteins regulated by the STAT1 inhibitor fludarabine (FLU). (**A**) Western blot analysis of mitophagy and lysosome molecule in *Immp2l*-deficient granulosa cells treated by STAT1 inhibitor fludarabine (FLU). (**B**) Detection of autophagic flux with the mCherry-GFP-LC3 lentivirus in *Immp2l*-deficient granulosa cells treated by STAT1 inhibitor fludarabine (FLU); at least 50 different cells in different fields were analyzed, and the dots were blindly counted by three different individuals. (**C**) Western blot analysis of UPR^mt^ and mitophagy marker molecules in *Immp2l*-deficient granulosa cells treated by HIF1α inhibitor 2-Methoxyestradiol (2-MeOE2). (**D**) Detection of the colocalization of UPR^mt^ molecules ATF4, ATF5, CHOP and Mito-tracker. (**E**) Detection of protein aggresome with PROTEOSTAT Aggresome Detection Kit in *Immp2l*-deficient granulosa cells treated by HIF1α inhibitor 2-Methoxyestradiol (2-MeOE2); at least 50 different cells in different fields were analyzed, and the dots were blindly counted by three different individuals. (* *p* < 0.05; ** *p* < 0.01; *** *p* < 0.001), (Scale bar: 10 μm).

**Figure 8:**
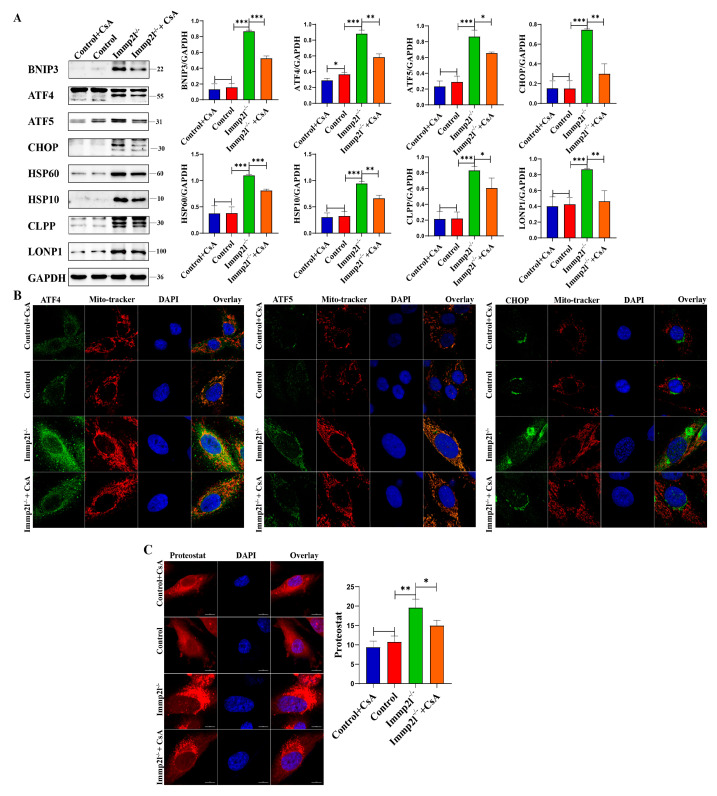
UPR^mt^ regulated by mitophagy inhibitor cyclosporin A (CsA) in *Immp2l*-deficient granulosa cells. (**A**) Western blot analysis of UPR^mt^ marker molecules in *Immp2l*-deficient granulosa cells treated by mitophagy inhibitor cyclosporin A (CsA). (**B**) Detection of the colocalization of UPR^mt^ molecules ATF4, ATF5, CHOP and Mito-tracker. (**C**) Detection of protein aggresome with PROTEOSTAT Aggresome Detection Kit in *Immp2l*-deficient granulosa cells treated by mitophagy inhibitor cyclosporin A (CsA); at least 50 different cells in different fields were analyzed, and the dots were blindly counted by three different individuals. (* *p* < 0.05; ** *p* < 0.01; *** *p* < 0.001), (scale bar: 10 μm).

The mitophagy markers HIF1α and BNIP3 were significantly decreased, but the lysosomal molecule Lamp2 was significantly increased, in STAT1-inhibited *Immp2l*^−/−^ granulosa cells compared with *Immp2l*^−/−^ granulosa cells (Figure 7A and Figure S4). These results were further confirmed by detecting autophagic flux, while the impairment of the autophagic flux was detected in *Immp2l*^−/−^ granulosa cells due to the impaired fusion of mitophagosomes and lysosomes, with the restoration of the autophagic flux attributed to the increased Lamp2 levels in STAT1-inhibited *Immp2l*^−/−^ granulosa cells (Figure 7B). HIF1α was reduced by the inhibitor 2-methoxyestradiol (2-MeOE2), and the mitophagy marker BNIP3 and UPR^mt^ markers ATF4, ATF5, CHOP, HSP60, HSP10, CLPP and LONP1 were significantly decreased in 2-MeOE2-treated Immp2l^−/−^ granulosa cells (Figure 7C), which was accompanied by decreases in ATF4, ATF5, and CHOP aggregates in mitochondria in 2-MeOE2-treated *Immp2l*^−/−^ granulosa cells (Figure 7D), as well as markedly reduced protein aggresomes in 2-MeOE2-treated *Immp2l*^−/−^ granulosa cells (Figure 7E). Mitophagy was reduced by the inhibitor cyclosporin A (CsA), which significantly decreased the mitophagy marker BNIP3. The UPR^mt^ markers ATF4, ATF5, CHOP, HSP60, HSP10, CLPP and LONP1 were significantly decreased by CsA treatment in *Immp2l*^−/−^ granulosa cells (Figure 8A), which was accompanied by decreases in ATF4, ATF5, and CHOP aggregation in mitochondria induced by CsA treatment in *Immp2l*^−/−^ granulosa cells (Figure 8B), as well as markedly reduced protein aggresomes in CsA-treated *Immp2l*^−/−^ granulosa cells (Figure 8C). Thus, mitophagy was regulated by STAT1 via the STAT1/HIF1α/BNIP3 pathway or STAT1/ATF4/HIF1α/BNIP3 pathway. The UPR^mt^ was also regulated by HIF1α/BNIP3-mediated mitophagy.

## 3. Discussion

Mitochondria are the multifunctional powerhouse of cells, including oocytes and granulosa cells. Compared to nuclear DNA, mtDNA is vulnerable to oxidative damage, then tends to mutations [26] and protein misfolding [27]. Mitochondrial dysfunction is one of the hallmarks of aging, and mitochondrial DNA (mtDNA) copy number and function decline with age in various tissues. There is increasing evidence to suggest that mitochondrial dysfunction drives aging, including ovarian aging.

Accelerated ovarian aging reduces female fertility and even causes infertility. Ovarian aging is triggered by several factors [28], such as mitochondrial dysfunction, excessive ROS production, endoplasmic reticulum stress, and autophagy. Under physiological conditions, ovarian function is controlled by mitochondrial homeostasis [19], and ovarian aging is triggered by mitochondrial dysfunction. Therefore, mitochondrial dysfunction and ROS are considered to be important in initiating ovarian aging and cell senescence.

As a nuclear gene encoding a mitochondrial inner membrane protein, Immp2l plays important roles in several biological processes. Behavior changed in the *Immp2l* knockdown or knockout mice, which is linked with autism [29]. The behavioral changes in *Immp2l* knockdown or knockout mice are associated with an antioxidant-like phenotype, but ROS levels were not increased and were significantly lowered in *Immp2l* knockout or knockout mice [30,31]. The *Immp2l* knockout mice had significantly less lean mass and overall body weight compared with wildtype littermates [30]. Immp2l plays an important role in maintaining ovarian function, and the deficiency of the *Immp2l* gene in mice causes infertility due to ovarian aging, which is caused by the cessation of ovarian follicle development in the secondary stage, as well as ovulation disorder [32]. Our previous study revealed that ovarian aging associated with *Immp2l* deficiency was mainly caused by granulosa cell senescence, and granulosa cell senescence and ovarian aging were delayed by treatment with the antioxidant melatonin [8]. Therefore, inhibiting the senescence of granulosa cells is the best choice for inhibiting aging in the ovary. Antioxidant treatment might be the best method to prevent *Immp2l* deficiency-caused age-dependent degeneration [33].

As an anthocyanidin, enocyanin might play important antioxidant and anti-inflammation roles. Indeed, granulosa cell senescence caused by *Immp2l* deficiency was alleviated by enocyanin treatment, which was accompanied by inhibitions of the senescence markers γH2AX, P16^INK4a^, IL-6 and IL-8. IL-6 and IL-8 are inflammation-related senescence markers and senescence-associated cytokines that induce self- and cross-reinforced senescence [34,35]. Therefore, enocyanin inhibits inflammation-related granulosa cell senescence. Cell senescence is triggered by excessive ROS production, and *Immp2l* deficiency increases ROS production. Our results reveal that ROS levels were reduced and MMP was increased in enocyanin-treated *Immp2l*^−/−^ cells compared with *Immp2l*^−/−^ cells, and enocyanin mitigated granulosa cell senescence, which was attributed to the reduction in ROS levels and improvements in mitochondrial function. In addition, the enocyanin belongs to flavonoids, and the flavonoids act as a potential redox balance mediator [36], improving the redox balance [37]. Redox regulates ROS production [38]. Therefore, enocyanin might recover the redox balance and mitochondrial function in *Immp2l*-deficient granulosa cells, and then reduce ROS production.

Mitochondria are the main source of ROS [39], and mitochondrial function is impaired by *Immp2l* deficiency, which might be attributed to an imbalance in mitochondrial proteostasis and mitochondrial homeostasis. Indeed, mitochondrial proteostasis was impaired in *Immp2l*-deficient granulosa cells due to the impaired UPR^mt^, leading to more unfolded and misfolded proteins aggregating in mitochondria. Then, the mitophagy/lysosome degradation system was activated to maintain mitochondrial proteostasis and mitochondrial homeostasis by clearing protein aggregates and organelles, such as mitochondria (i.e., mitophagy) [40]. In *Immp2l*-deficient cells, HIF1α-BNIP3-mediated mitophagy is activated to clear protein aggregates and organelles, but impairs mitophagosome–lysosome fusion, which is accompanied by autophagosome accumulation in senescent granulosa cells due to reduced Lamp1 and Lamp2 protein levels [24,41,42]. The HIF1α-BNIP3–mitophagy pathway is impaired in the ovarian granulosa cells of PCOS rats [24], and restoring the balance of the HIF1α-BNIP3–mitophagy pathway might be a novel therapeutic strategy for PCOS. Therefore, restoring the balance of HIF1α-BNIP3–mitophagy and lysosomes might be a novel mechanism by which enocyanin alleviates granulosa cell senescence. Indeed, mitophagy controls the mitochondrial quality through removing impaired/dysfunctional mitochondria to maintain redox homeostasis and sustain cell viability [43]. Our results suggest that the balance of HIF1α-BNIP3–mitophagy and lysosomes was restored by enocyanin treatment, which was accompanied by a marked decrease in cell senescence markers and ROS. Thus, enocyanin delays granulosa cell senescence by maintaining the balance of the HIF1α-BNIP3–mitophagy pathway and redox homeostasis.

Interestingly, the STAT1 protein, which is closely related to oxidative stress and ROS, was markedly increased in *Immp2l*-knockdown granulosa cells, and was mostly regulated by the S-glutathionylation [5,6] and S-nitrosylation [7] of redox-related proteins. Our results suggest that STAT1 did not translocate into the nucleus and accumulate in mitochondria; it is not controlled by phosphorylation, but is controlled by the posttranslational modifications S-glutathionylation and S-nitrosylation. Furthermore, S-glutathionylation and S-nitrosylation were regulated by enocyanin treatment. Therefore, the STAT1 protein was regulated by enocyanin, which controlled S-glutathionylation and S-nitrosylation. The modification sites for S-glutathionylation and S-nitrosylation on STAT1 need to be explored in further studies. Additionally, S-glutathionylation and S-nitrosylation are redox-mediated modifications that regulate protein function [44]. *Immp2l* deficiency causes imbalances in mitochondria-associated redox status [45], and redox regulates the STAT1 [4].

In addition, UPR^mt^ and mitophagy were regulated by STAT1 inhibition in *Immp2l*-deficient granulosa cells.

ATF4 is a core molecule in UPR^mt^ [20]; UPR^mt^ is initiated by ATF4, and ATF4 is downstream of STAT1 [46]. ATF4 was downregulated by inhibiting STAT1 in the context of *Immp2l* deficiency-induced granulosa cell senescence, and the co-IP results reveal that STAT1 targeted the ATF4 protein. Thus, *Immp2l* deficiency impairs the UPR^mt^ via STAT1/ATF4, and enocyanin improves UPR^mt^ by regulating the STAT1/ATF4 pathway.

**Figure 9:**
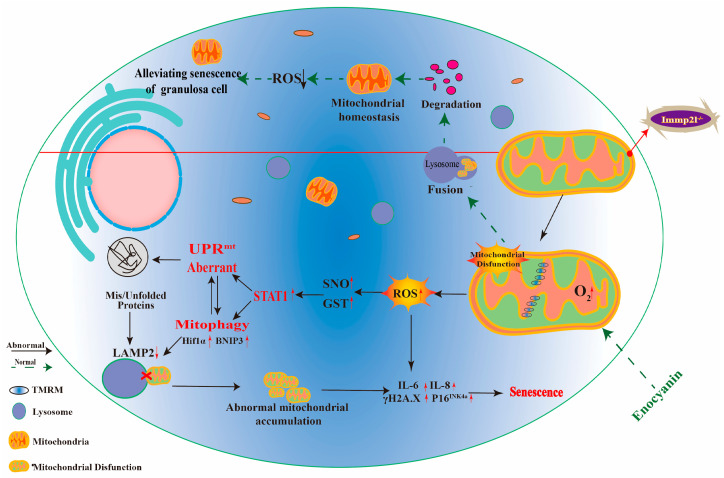
The pathway by which enocyanin alleviates *Immp2l* deficiency-induced granulosa cell senescence. Under normal physiological conditions, mitochondrial proteostasis and mitochondrial homeostasis in granulosa cells are maintained by the UPR^mt^ and mitophagy. However, *Immp2l* deficiency in granulosa cells impairs the UPR^mt^ and mitophagy, leading to excessive unfolded and misfolded protein aggregation in mitochondria, dysfunctional mitochondria aggregation in cells, and excessive aggregated dysfunctional mitochondria producing excessive ROS, thereby triggering granulosa cell senescence. Enocyanin treatment alleviates granulosa cell senescence and improves the function of UPR^mt^ and mitophagy through STAT1/ATF4-mediated UPR^mt^ and STAT1/(ATF4)/HIF1α/BNIP3-mediated mitophagy, restoring mitochondrial proteostasis and mitochondrial homeostasis in granulosa cells and reducing ROS production.

Additionally, ATF4 targets HIF1α [47,48]. The co-IP results reveal that ATF4 targeted HIF1α, STAT1 targeted HIF1α, and HIF1α targeted BNIP3. Thus, *Immp2l* deficiency activates mitophagy via STAT1/ATF4/HIF1α/BNIP3 or STAT1/HIF1α/BNIP3, and enocyanin improves mitophagy by regulating the STAT1/ATF4/HIF1α/BNIP3 or STAT1/HIF1α/BNIP3 pathway (Figure 9). Inhibiting HIF1α with 2MeOE2 and inhibiting mitophagy with CsA further confirmed that the HIF1α/BNIP3 pathway was activated in *Immp2l* deficiency-induced granulosa cell senescence, and that HIF1α/BNIP3 was regulated by STAT1. Conversely, the UPR^mt^ was regulated by HIF1α/BNIP3-mediated mitophagy.

Overall, enocyanin delays granulosa cell senescence induced by *Immp2l* deficiency through STAT1-mediated mitophagy and UPR^mt^, and STAT1 is regulated by enocyanin-controlled posttranslational modifications of S-glutathionylation and S-nitrosylation (Figure 9).

## 4. Materials and Methods

### 4.1. Mice and Cells

*Immp2l* gene-knockout mice were purchased from Cyagen Biosciences Inc. (Suzhou, China). The 21- to 26-day-old female mice were purchased and bred in the Laboratory Animal Center at Ningxia Medical University and were maintained at 24 ± 2 °C in a light-controlled room (12 h light:12 h darkness) with free access to food and water. The animal procedures strictly followed the US guidelines of the National Institutes of Health and were approved by the Institutional Animal Care and Use Committee at Ningxia Medical University (2021-Z011). All animals were operated on under sodium pentobarbital anesthesia, and all efforts were made to minimize pain and discomfort.

Primary granulosa cells from mice were isolated and cultured in vitro according to our previous studies [8]. The human granulosa tumor cell line KGN maintains most physiological activities of immature granulosa cells, including steroidogenesis, such as AMH, and apoptosis [49,50,51]; therefore, it is a very widely used and acceptable cell line for exploring granulosa cell function. Correspondingly, our previous study suggested that *Immp2l* gene deficiency causes ovarian follicle developmental arrest in the secondary stage, attributable to granulosa cell senescence [8]. Therefore, KGNs are optimal cells to explore the role of *Immp2l* in granulosa cell. KGN cells were purchased from Cyagen, the *Immp2l* gene was knocked out in the KGN cell line via the CRISPR/Cas9 method by Cyagen (Suzhou, China), and the procedure of knocking out *Immp2l* in the KGN cell line is briefly described in the Supplemental Materials (Figures S5–S7), while the siRNA sequence of *STAT1* and *Immp2l* is shown in Table S1. The granulosa cells were treated with 10 mM enocyanin (HY-114336, MCE, grape-skin extract, GSE) according to previous studies [52,53] with minor modifications; after 24 h, the treated granulosa cells were collected for further analysis.

### 4.2. Western Blot Analysis

Western blot analysis was performed according to our previous study [8]. The information on antibodies is shown in Table S2. GAPDH was used as a reference protein, and the protein bands were analyzed using ImageJ software (V1.8.0.112).

### 4.3. Coimmunoprecipitation

The coimmunoprecipitation (co-IP) procedure was performed according to our previous study [8]. The antibodies used for co-IP were the same as those described for Western blotting.

### 4.4. Immunofluorescence Analysis

The immunofluorescence procedure was performed as described in our previous study [8]. The primary antibodies used for Western blotting were used for these experiments, and goat anti-rabbit FITC and goat anti-mouse TRITC secondary antibodies were purchased from ZSGB-BIO (Beijing, China). MitoSOX™ Red mitochondrial superoxide indicator (M36008) was purchased from Invitrogen (Waltham, MA, USA). TMRM (I34361) was purchased from Thermo Fisher (Waltham, MA, USA). HBAD-mRFP-GFP-LC3 (HBAD-1006) was purchased from HANBIO (Shanghai, China). The procedure of staining with the fluorescent probe was performed according to the manufacturer’s instructions. The images were obtained with confocal laser scanning microscopy (CLSM), and the fluorescence intensity was analyzed using ImageJ software.

### 4.5. Mitochondrial Membrane Potential and Mitochondrial Superoxide Assays

Mitochondrial membrane potential (MMP) was determined in cells for ex vivo analysis using the potentiometric probe TMRM (Molecular Probes, Life Technologies, Carlsbad, CA, USA). After surface-staining, the cells were incubated with TMRM (100 nM) for 15 min at 37 °C and photographed with a confocal laser scanning microscope. Images were obtained from different parts of the cells and analyzed using ImageJ software.

Mitochondrial superoxide levels were measured in cells and were incubated with MitoSOX Red dye (5 μm; Thermo Fisher) for 15 min at 37 °C according to the manufacturer’s protocol, and images were obtained by confocal laser scanning microscopy (CLSM). Images were obtained from different parts of the cells and analyzed using ImageJ software.

### 4.6. Detection of Senescent Cells

The Invitrogen™ CellEvent™ Senescence Green Detection Kit (Invitrogen, Waltham, MA, USA) and Senescence β-Galactosidase Staining Kit (KeyGEN, Nanjing, China) were used to stain the senescent cells, and the fluorescence intensity was analyzed by ImageJ software.

### 4.7. Protein Aggregation Assay

KGN cells were seeded at a density of 2 × 10^5^/well in 12-well cell culture plates (Corning, Corning, NY, USA) containing glass coverslips, and aggregated proteins were detected using the PROTEOSTAT Aggresome Detection Kit (ENZ-51035; Enzo Life Sciences, Farmingdale, NY, USA) according to the manufacturer’s instructions. Briefly, cells were seeded in 12-well culture plates, fixed with 4% formaldehyde at 25 °C for 30 min and permeabilized with 0.5% Triton X-100 and 3 mM EDTA (pH 8.0) on ice for 30 min. Subsequently, the cells were stained with a dual detection reagent containing Hoechst 33258 (nuclear staining) and PROTEOSTAT dye reagent and then incubated at 25 °C for 30 min. Aggregated protein imaging was performed using confocal laser scanning microscopy (CLSM). Images were obtained from different parts of the cells and analyzed using ImageJ software.

### 4.8. Statistical Analysis

All experiments were repeated at least thrice in triplicates each time. The data are presented as the mean ± SEM. Data from more than two groups were analyzed with One-way ANOVA followed by Fisher’s least significant difference test, and data from two groups were analyzed with *t* tests using SPSS software (version 21.0; SPSS, Inc., Chicago, IL, USA). Differences were considered significant at *p* < 0.05.

## Data Availability

The original contributions presented in the study are included in the article/Supplementary Materials, and further inquiries can be directed to the corresponding authors.

## Supplementary Materials

The following supporting information can be downloaded at: https://www.mdpi.com/article/10.3390/ijms252011122/s1.
