# Supplementary material for: Immp2l Deficiency Induced Granulosa Cell Senescence Through STAT1/ATF4 Mediated UPRmt and STAT1/(ATF4)/HIF1α/BNIP3 Mediated Mitophagy: Prevented by Enocyanin
Source: Int J Mol Sci. 2024 Oct 16;25(20):11122. doi: 10.3390/ijms252011122 (PMC11508440; doi:10.3390/ijms252011122)
Supplement: Supplementary file 1 [file ijms-25-11122-s001.zip › ijms-3227559-supplementary.pdf]

Supplementary Materials

Table S1. siRNA sequence.

Gene name		Sequence (5′–3′)
Negative control	Sense	5′-UUCUCCGAACGUGUCACGUTT-3′
	Antisense	5′-ACGUGACACGUUCGGAGAATT-3′
STAT1 (human)	Sense	5′-CCUACGAACAUGACCCUAUTT-3′
	Antisense	5′-AUAGGGUCAUGUUCGUAGGTT-3′
Immp2l (mouse)	Sense	5′-CUCCAGACUGGUGAGAAAUTT-3′
	Antisense	5′-AUUUCUCACCAGUCUGGAGUG-3′

Table S2. Western blot antibody.

Antibody	Cat No.	Reagent brand
GAPDH	60004-1-Ig	Proteintech
Immp2l	15970-1-AP	Proteintech
IL-6	66146-1-Ig	Proteintech
IL-8	27095-1-AP	Proteintech
$P16^{INK4a}$	10883-1-AP	Proteintech
HIF1α	20960-1-AP	Proteintech
BNIP3	68091-1-Ig	Proteintech
LAMP2	66301-1-Ig	Proteintech
ATF4	10835-1-AP	Proteintech
ATF4	60035-1-Ig	Proteintech
CHOP	15204-1-AP	Proteintech
CLPP	15698-1-AP	Proteintech
LONP1	66043-1-Ig	Proteintech
STAT1	10144-1-AP	Proteintech
HSP60	66041-1-Ig	Proteintech
IL-6	ab259341	Abcam
$P16^{INK4a}$	ab211542	Abcam
$\gamma H_2A.X$	ab81299	Abcam
HIF1α	ab179483	Abcam
ATF5	ab184923	Abcam
Galectin3	ab209344	Abcam
HSP10	abs130120	absin
IL-8	A12452	Abclonal
γH2A.X	A11412	Abclonal
BNIP3	44060s	Cell Signaling Technology
Phospho-STAT1(Tyr701)	9167	Cell Signaling Technology
Phospho-STAT1(Ser727)	9177	Cell Signaling Technology
Normal Rabbit IgG	2729S	Cell Signaling Technology
LAMP1	Sc-17768	Santa Cruz Biotechnology
Anti-Glutathione	101-A	VIROGEN
Anti-S-Nitroso-Cys	SNOBSA-N-100	Alpha Diagnostic International
Anti-Rabbit-IgG/Anti-mouse-IgG		Cell Signaling Technology

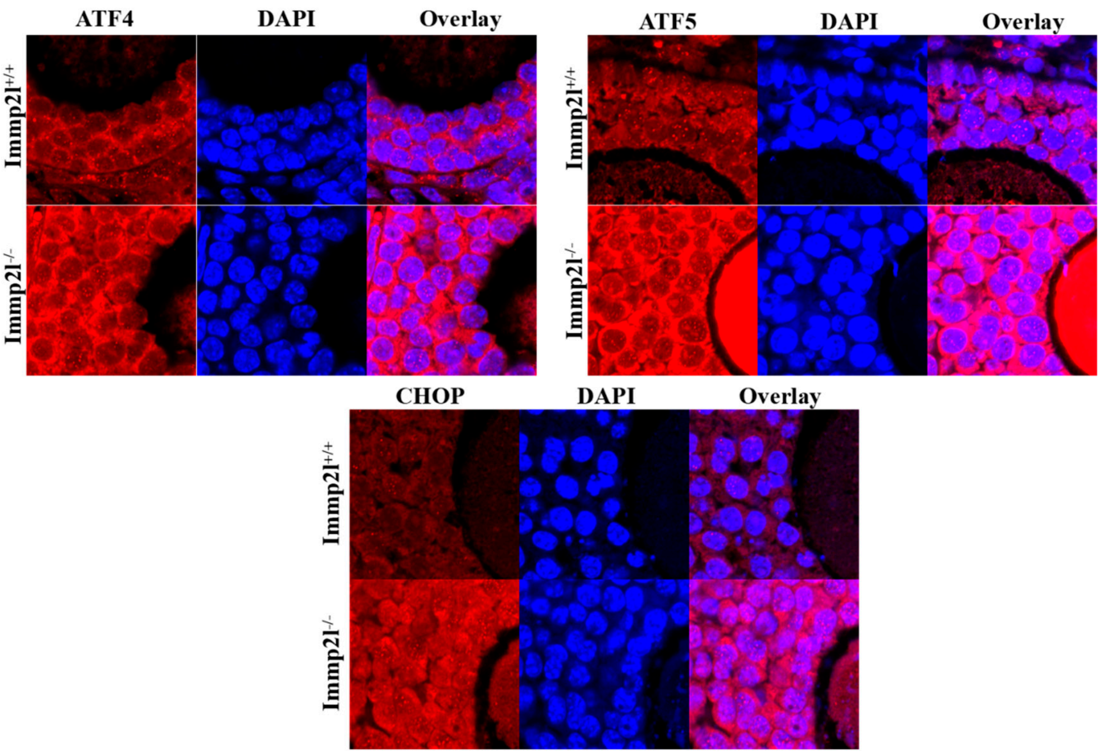

Figure S1. The Impairment of UPR^{mt} attributed to ATF4, ATF5 and CHOP aggregated in the cytoplasm but not translocated into nucleus. The localization of UPR^{mt} core molecules ATF4, ATF5 and CHOP in in secondary follicles in vivo in Immp2l$^{+/+}$ and Immp2l$^{-/-}$ mice.

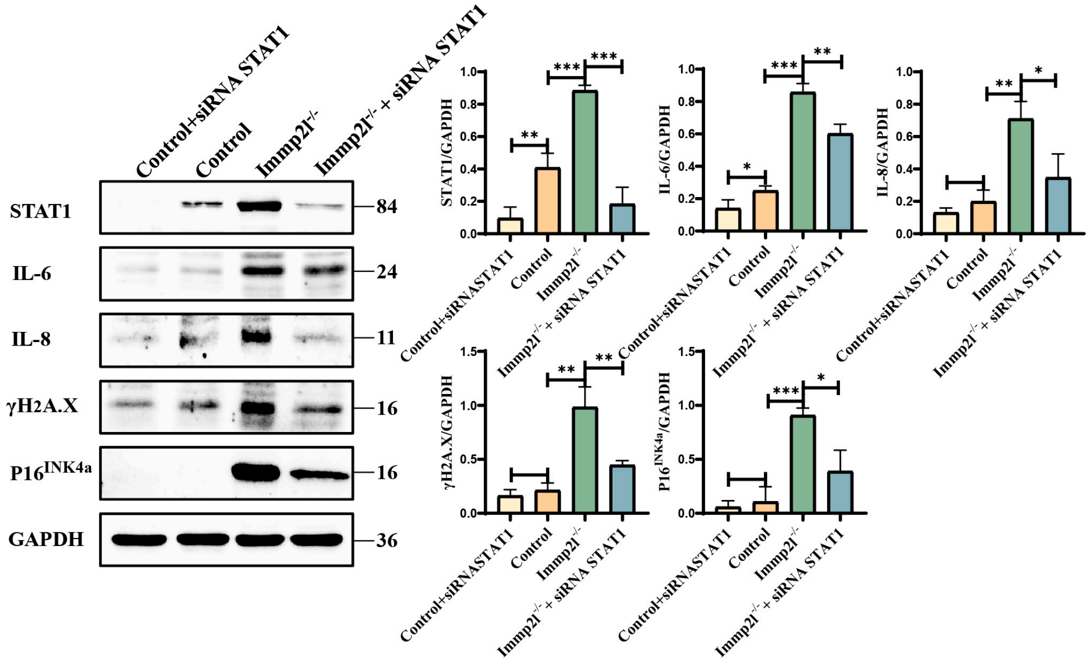

Figure S2. Inhibit STAT1 by siRNA alleviates Immp2l deficient induced granulosa cell senescence. Western blot analysis of cell senescence marker molecules in Immp2l-deficient granulosa cells treated with the STAT1 siRNA. (*$P < 0.05$; **$P < 0.01$; *** $P < 0.001$).

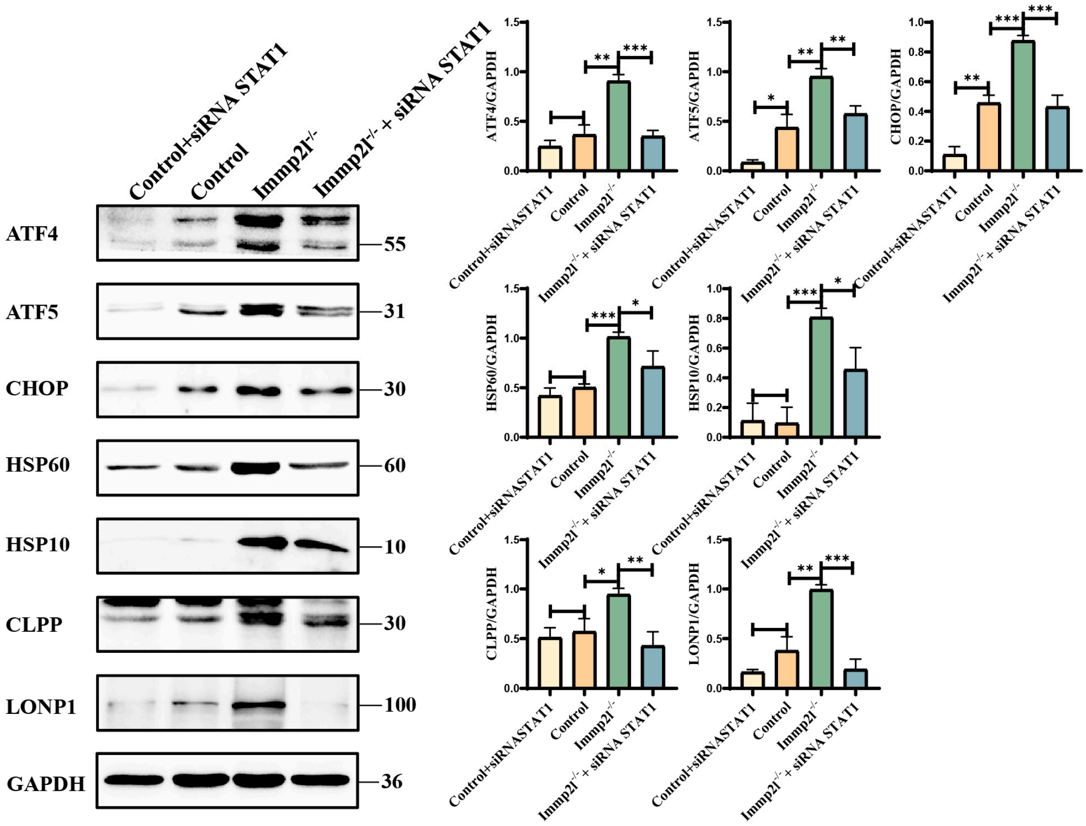

Figure S3. Inhibit STAT1 by siRNA restore Immp2l deficient induced UPR^{mt} dysfunction. Western blot analysis of UPR^{mt} marker molecules in Immp2l-deficient granulosa cells treated with the STAT1 siRNA. (*$P < 0.05$; **$P < 0.01$; *** $P < 0.001$).

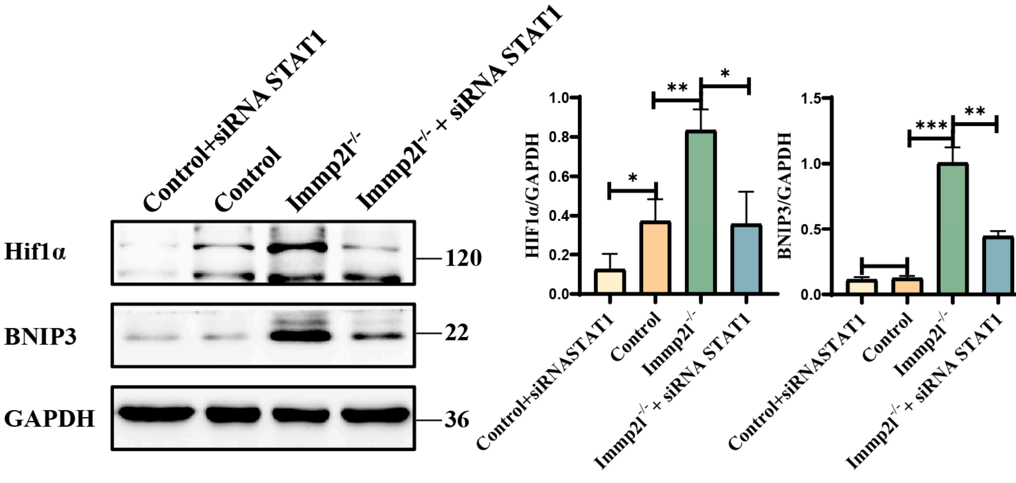

Figure S4. Inhibit STAT1 by siRNA restore Immp2l deficient induced mitophagy impairment. Western blot analysis of mitophagy marker molecules in Immp2l-deficient granulosa cells treated with the STAT1 siRNA. (*$P < 0.05$; **$P < 0.01$; *** $P < 0.001$).

1. The transfection of granulosa cells

The The gRNA sequence of human Immp2l gene was used as AGCCTTCTTTGAATCCTGGG-GGG, the lentivirus vectors were constructed and transfected into 293T cells, then lentivirus particles were collected and transfected into KGN cells (KGN cell was proven without pollution and mycoplasma, as showed in Figure S5).

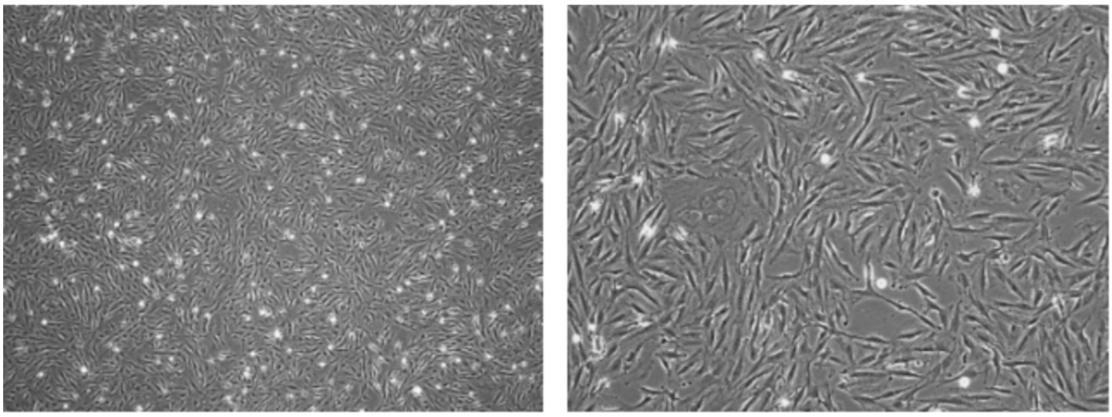

Figure S5. KGN cells.

2. The identify of Immp2l knockout cells

The signal cell was cloned (Figure S6), and identified by sequencing, and results showed in Figure S7.

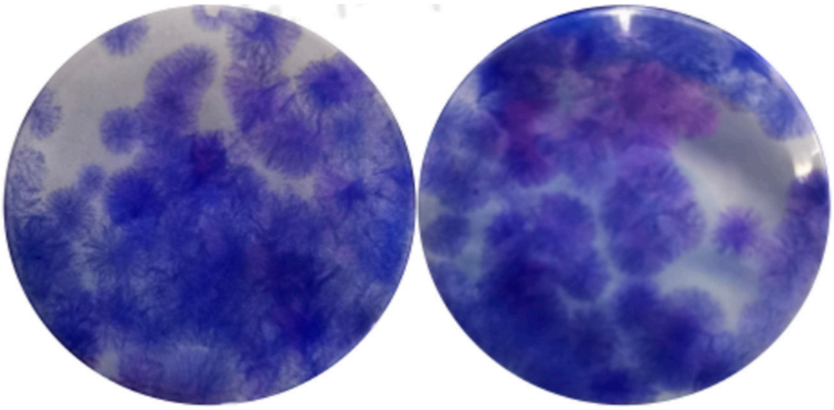

Figure S6. The percentage of cell clone.

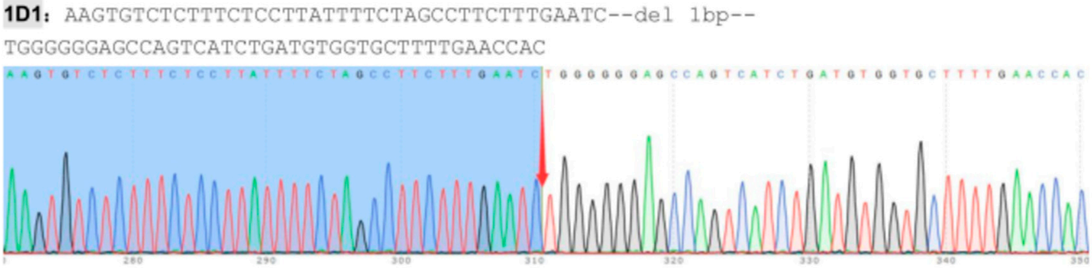

Figure S7. The sequencing results of Immp2l gene knockout cells .
